# Achieving herd immunity in South America

**DOI:** 10.1186/s41256-023-00286-2

**Published:** 2023-02-01

**Authors:** Don Eliseo Lucero-Prisno, Deborah Oluwaseun Shomuyiwa, Creuza Rachel Vicente, María José González Méndez, Shohra Qaderi, Jaifred Christian Lopez, Yidnekachew Girma Mogessie, Jason Alacapa, Lila Chamlagai, Remy Ndayizeye, Pelin Kinay

**Affiliations:** 1 Department of Global Health and Development, London School of Hygiene and Tropical Medicine, London, UK; 2 Faculty of Management and Development Studies, University of the Philippines Open University, Los Banos, Laguna Philippines; 3 Faculty of Public Health, Mahidol University, Bangkok, Thailand; 4 Faculty of Pharmacy, University of Lagos, Lagos, Nigeria; 5 Department of Social Medicine, Federal University of Espírito Santo, Vitória, Brazil; 6 Postgraduate Program in Infectious Diseases, Federal University of Espírito Santo, Vitória, Brazil; 7 Global Health Focus South America, Santiago, Chile; 8 Maternal Fetal Care Center, Boston Children’s Hospital, Harvard Medical School, Boston, MA USA; 9 Department of Population Health Sciences, School of Medicine, Duke University, Durham, NC USA; 10 Johns Hopkins Bloomberg School of Public Health, Baltimore, MD USA; 11 Johns Hopkins Carey Business School, Baltimore, MD USA; 12 School of Public Health, Brown University, Providence, RI USA; 13 George Washington University, Washington, DC USA; 14 School of Climate Change and Adaptation, University of Prince Edward Island, Charlottetown, Canada

**Keywords:** Herd immunity, COVID-19, Vaccination, Population health, South America, Pandemic

## Abstract

South America, once an epicenter of COVID-19, has stayed on the road of continued management of the pandemic. The region initially struggled to cope with the pandemic as it experienced spiraling numbers of infections and overwhelmed public health systems. South America has risen in its pandemic response to be the region with the highest global vaccination rate. The region posed a strong vaccination drive, with over 76% of its population fully vaccinated with the initial protocol. South America leveraged its deeply rooted vaccination culture and public health confidence among its population. Herd immunity is an integral concept in population infectious disease management. Attaining herd immunity is presently not feasible with available vaccines, but the high vaccination rate in the region depicts the acceptance of vaccination as a strategy for population protection. The availability of effective transmission-blocking vaccines, the continuous implementation of strategies that will enable the undisrupted supply of the vaccines, equity in access to the vaccines, improved vaccine acceptance, and trust in the vaccination and public health systems will help shepherd the region towards herd immunity. Local vaccine production backed with investment in infrastructure and international collaboration for research and knowledge development will also drive population safety.

## Background

COVID-19 reached South America later than other regions; however, when it reached its shores, the region became an epicenter of the COVID-19 pandemic [1]. The largest country in South America, Brazil, became the most affected country with over 34 million cases by September 2022 [2]. As of September 25, 2022, South America has recorded more than 63 million cases and a growing number of fatal COVID-19 cases, which became the third highest, after Europe and South-East Asia [3]. The under-resourced public health systems of the region's low and middle-income countries (LMICs) struggled to cope with the pandemic.

The Coronavirus disease, officially called COVID-19, is caused by a novel virus called the SARS-CoV-2 coronavirus. COVID-19 is one of the biggest public health conundrums of the twenty-first century, with over 500 million cases and more than 6.2 million deaths globally [2]. It continues to impact countries in the world with new waves and variants [4]. With the start of 2022, countries in South America reported 6.1 million new cases of COVID-19—a 250% increase from the same period in 2021. Even with increased vaccination in the region and stable number of COVID-19 deaths, increased emergency visits and hospitalizations culminated in challenges in the pandemic response [5]. Peru had the region's highest recorded COVID-19 mortality rate at 5.4%, followed by Ecuador (3.7%), Paraguay (2.7%), Colombia (2.2%) and Brazil (2%), since September 2022 [6]. Peru was confirmed to have the highest global death rate from COVID-19, with a death toll of over 185,000 out of 33 million people infected [7].

The spike in COVID-19-related mortality is linked to health systems which are under pressure and underfunded, entrenched social inequalities, inconsistent regional vaccination rates, and high transmission rates of the novel COVID-19 variants [8]. The effectiveness of available vaccines and the phenotypic instability of COVID-19 ensure that reaching herd immunity by increasing vaccination numbers may be an elusive goal. This article aims to assess the COVID-19 development in South America, the region's health systems' response to building population immunity, and recommendations to drive herd immunity in South America.

## Vaccination efforts in South America

**Fig. 1:**
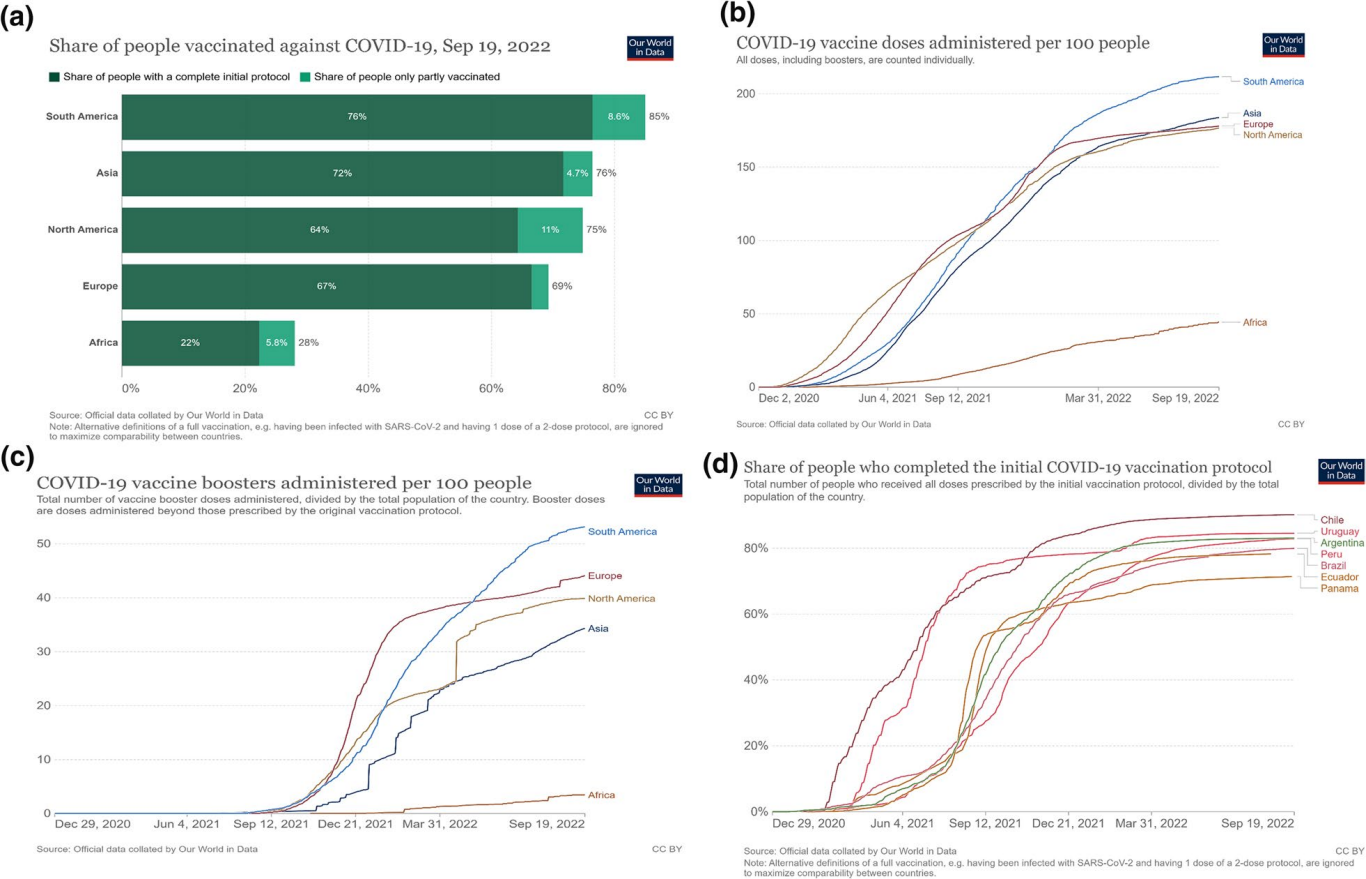
**a** Share of population vaccinated against COVID-19. *Source*: Edouard Mathieu, Hannah Ritchie, Lucas Rodés-Guirao, Cameron Appel, Charlie Giattino, Joe Hasell, Bobbie Macdonald, Saloni Dattani, Diana Beltekian, Esteban Ortiz-Ospina and Max Roser (2020)—"Coronavirus Pandemic (COVID-19)". Published online at OurWorldInData.org. Retrieved from: 'https://ourworldindata.org/coronavirus' [Online Resource]. **b** COVID-19 vaccine doses administered per 100 people. *Source*: Edouard Mathieu, Hannah Ritchie, Lucas Rodés-Guirao, Cameron Appel, Charlie Giattino, Joe Hasell, Bobbie Macdonald, Saloni Dattani, Diana Beltekian, Esteban Ortiz-Ospina and Max Roser (2020)—"Coronavirus Pandemic (COVID-19)". Published online at OurWorldInData.org. Retrieved from: 'https://ourworldindata.org/coronavirus' [Online Resource]. **c** COVID-19 vaccine boosters administered per 100 people. *Source*: Edouard Mathieu, Hannah Ritchie, Lucas Rodés-Guirao, Cameron Appel, Charlie Giattino, Joe Hasell, Bobbie Macdonald, Saloni Dattani, Diana Beltekian, Esteban Ortiz-Ospina and Max Roser (2020)—"Coronavirus Pandemic (COVID-19)". Published online at OurWorldInData.org. Retrieved from: 'https://ourworldindata.org/coronavirus' [Online Resource]. **d** Share of population who completed the initial COVID-19 vaccination protocol in South American countries. *Source*: Edouard Mathieu, Hannah Ritchie, Lucas Rodés-Guirao, Cameron Appel, Charlie Giattino, Joe Hasell, Bobbie Macdonald, Saloni Dattani, Diana Beltekian, Esteban Ortiz-Ospina and Max Roser (2020)—"Coronavirus Pandemic (COVID-19)". Published online at OurWorldInData.org. Retrieved from: 'https://ourworldindata.org/coronavirus' [Online Resource]

As the world moves on with dealing with the pandemic through vaccination, South America is working to keep the pace despite being designated as the region with the greatest global inequality. With 30% of the region living under the poverty line, and 60% employed in the informal sector, there was an increased risk of rapid virus transmission and limited access to care among the disadvantaged population [9]. Evidence suggests that COVID-19 vaccines protect against serious illness, hospitalization, and death [10]. The global COVID-19 vaccination has increased, with over 5 billion people receiving at least one dose of the COVID-19 vaccine [2]. South America has had 76.9% of its population fully vaccinated with the initial vaccine protocol (Fig. 1a). Despite the slow start to vaccination, South America represents a region with one of the highest vaccination rates globally (Fig. 1b, c). Chile has the highest vaccination rate of 90% as of September 2022 (Fig. 1d). Asia and Europe vaccinated 72% and 67% for the initial protocol [11]. Of the over 12.68 billion vaccine doses administered globally, about 8% of the vaccine doses have been administered in the region [11]. This accounts for about 923.94 million doses, including booster doses.

South America's vaccine success story is attributed to several factors. It may vary from country to country, but it can be traced to a historically deep-rooted culture of vaccination [12] and respect for public health systems that still serve the population, however fragile and weakened they became during the pandemic [13]. Even with uneven containment measures and largely lax government influence, South America has risen in vaccine acceptance [14]. The vaccination process has not been without challenges. The emergence of different virus variants has impacted vaccination. As of February 2022, the Alpha, Beta, Gamma, Delta, Omicron, and even the sub-variants of the Omicron have been discovered. The emergence of these virus variants and the need for booster shots have posed a significant challenge to the goal of herd immunity.

The timeline for the research, discovery and development of vaccines usually takes years. However, the dire need to address the COVID-19 pandemic and the funding for the development of current vaccines has shifted the time curve. This facilitated the production of safe and effective vaccines to prevent and control the virus spread in record time. Nearly two years after the first case was reported, the virus has been managed symptomatically with antiviral medications, vaccines, COVID-19-related diagnostics, and adequate research and development. Several COVID-19 vaccines have been validated for use globally by the World Health Organization (WHO) to ensure that they meet quality, safety, and efficacy standards. As of May 16, 2022, ten vaccines have received this emergency authorization in the region [11]. These vaccines have been approved for use in several countries, and vaccination programs are well underway. Leading vaccines in the region are AstraZeneca-Vazevira, Pfizer BioNTech—Comirnaty and SII- Covishield [2]. Other vaccines in use include Sinovac-CoronaVac, Moderna-Spikevax, Bharat-Covaxin, Sinopharm BIBP COVID-19 vaccine, Cansino-Convidecia, Sputnik V vaccine, and Juplhar-Hayat-Vax. Most vaccines in South America require two doses, but the CanSino vaccines are single doses. Brazil started its vaccine rollout in January 2021 with the domestically produced vaccine Sinovac and Covishield, while Argentina administered Russian-made Sputnik V from December 2020 [15]. Sputnik V, the Russian vaccine that first promised safety for the South American countries, proved short-lived due to production delays leading to shortages and prolonged waiting periods. Pan American Health Organization (PAHO) data attributed the surge in the vaccination rate in South America to the accelerated delivery of European, American, Chinese, and homegrown vaccines that several South American countries received [16].

As the COVID-19 battle continues, allocation and access to vaccines are the mainstays of arguments. COVAX, an international alliance co-led by the Coalition for Epidemic Preparedness Innovations (CEPI), Global Alliance for Vaccines and Immunization (GAVI) and the WHO, strives to promote fair and equitable access to the COVID-19 vaccine worldwide [17]. The PAHO Revolving Fund served as the region’s recognized procurement agent for COVAX. UNICEF, a key delivery partner, provided support for the receipt and administration of the vaccines [18]. As of January 20, 2022, PAHO reported that over 96 million doses had been delivered or were in transit through COVAX, with over 26 million doses provided through country donations, including from the United States [19]. Mexico is forecasted to have received the largest vaccination allocation, having secured over 35 million vaccines via the COVAX initiative [16]. To improve vaccine supply in the region, PAHO's Revolving Fund, which has purchased and supplied almost 100 million doses to 33 countries so far, is also on track to obtain a further 200 million doses on behalf of the region in 2022 [17]. China donated vaccines to selected countries–100,000 Sinopharm doses to Bolivia and 500,000 Sinopharm doses to Venezuela [18].

The Fair Allocation Network, advised by WHO, was set up for faster and more efficient containment of the pandemic and more equitable distribution of vaccines to help stop the pandemic's acute phase and progress towards attaining herd immunity [19]. PAHO coordinated deliveries with other COVAX partners, such as UNICEF, CEPI, WHO, and GAVI, the Vaccine Alliance, to support international logistics and ensure safe and prompt delivery of the vaccines. An initiative for additional access to COVID-19 vaccines was launched in the region by entering into long-term agreements with selected manufacturers and biomedical centers in Argentina and Brazil to produce vaccines [6].

## Herd immunity in South America

Herd immunity is an essential concept in reducing the unmitigated spread of infection. Herd immunity, also known as "population immunity", involves protecting a population from infectious diseases. This form of immunity provides indirect protection to a susceptible population. This protection can be through vaccination or natural immunity from the previous infection [20]. Vaccination is the preferred method due to the high risk of death from natural infection [20]. With herd immunity, the population has a high level of immunity to infection as there is reduced individual susceptibility to infection. Herd immunity is vital in protecting vulnerable groups [21]. The herd immunity threshold is the minimum proportion of the population that must be immune to an infectious disease, usually due to vaccination, for the incidence of the disease to remain stable or decrease [22]. This threshold depends on the disease's infectiousness and the effectiveness of available vaccines [23].

Throughout the pandemic and with the emergence of vaccines, there have been global speculations on the required vaccinated population number for herd immunity. WHO has indicated that herd immunity against COVID-19 is attained when 60–70% of a population is immune. However, some medical experts have suggested that the figure is higher due to the high transmission rate of the virus [24]. As herd immunity cannot be reached through COVID-19 infection, effective vaccination presents a safe strategy for attaining population immunity. The natural infection has a high mortality cost [24]. South America has a population of over 400 million and is equivalent to about 5.53% of the world’s population [2].

## Challenges in attaining herd immunity in South America

Globally, the herd immunity concept and its thresholds have been questioned in attempts to control or eliminate infectious diseases. Attaining herd immunity through vaccination entails ensuring vaccine effectiveness in curtailing disease transmission, securing an adequate number of effective vaccines, facilitating easy and convenient access to these vaccines; in the case of high vaccination rate as seen in the region, maintaining vaccine confidence and evading vaccine complacency. While the South American region is forging ahead in the vaccination process and ranking high in vaccination rates, attaining a COVID-19 herd immunity level still looks far off the mark. High vaccination numbers do not seem to translate to developing herd immunity for COVID-19.

The herd immunity threshold is reached under the assumption that the immune population get permanent protection from the different variants of the COVID-19 diseases and does not further transmit. This is synonymous with eliminating viral diseases. Infectious diseases have proven to be tasking in their elimination. In the history of humans combating infectious diseases, only Smallpox has been eliminated [21]. This required administration of effective vaccines, 184 years of dedicated action, the presentation of pronounced symptoms, and low rates of reinfection. COVID-19 can be transmitted asymptomatically and without indicative signs [25]. High rates of reinfections and the presence of non-human reservoirs make COVID-19 immunity complicated.

Herd immunity with the available COVID-19 vaccines is complicated as they do not wholly block the virus's transmissibility. Available vaccines have been indicated to reduce hospitalizations by reducing the severity of symptoms upon infection with minimal action on transmission-blocking [21]. Vaccines that effectively block transmission are currently not available. The lack of substantial engagement with the systemic immune system ensures that infection and available vaccines fail to induce prolonged protection in the population [21]. Herd immunity will be challenging to attain because even if a majority of the population may be fully vaccinated. Fading immunity will mean that a large group will not remain immune for an extended period.

New variants like the Omicron and its subvariants pose a significant challenge to herd immunity. Despite an estimated 76% of the population being infected in Manaus, Brazil, the herd immunity threshold appears to have not been met [24]. The inability to reach herd immunity despite widespread infection in Manaus and other locations worldwide is due to a mix of waning immunity and new variants. Current vaccines have had minimal impact on transmission due to waning immunity and immune-evasive variants. The formidable challenge of the new variants is their higher transmissibility and immune escape [21]. This makes it challenging to adequately estimate the threshold for attaining herd immunity.

COVID-19 vaccination in South America is influenced by social disparities such as economic status and global geographical inequalities in vaccine distribution [8]. Even with the high vaccination rate in the region, vaccine inequity is an issue that continues to divide the region and slows the achievement of herd immunity. Vaccine inequity is present within and between countries. A disparity in immunization rates between the countries shows a clear divide. Chile, the region's most vaccinated country, has a vaccination rate three times that of French Guiana [2]. Low population density or weak state presence in territories like Guiana and the Falkland Islands have substantially lower immunization rates. Globally, of the 12.7 billion vaccines administered and 62.5% of the global population that is fully vaccinated as of September 2022, only 22% and 59% of people in low-income countries and lower-middle-income countries, respectively, have been fully vaccinated, a massive difference from 81 to 85% of the high income and upper-middle-income countries respectively [11].

## Recommendations

- Transmission-blocking vaccines

Herd immunity is only attainable if transmission-blocking vaccinations or complete population vaccinations are available [26]. Effective vaccines with lasting protection are essential. Intra-nasal vaccines currently in development may offer such protection [27]. Vaccine effectiveness impacts the potential for reaching herd immunity [28]. Present vaccination strategies have been integral for improving recovery and reducing mortality; future transmission-blocking vaccines will forge the path to herd immunity. The rollout of the updated bivalent boosters and vaccines from Moderna and Pfizer-BioNTech also presents the potential to curb COVID-19 transmission. Newer and more effective vaccines, such as intranasal and inhaled, will offer a new push for transmission prevention. Safe and effective vaccines approved for use in all ages will increase vaccination levels and improve the chances of herd immunity.

b. Vaccine Uptake

Sustained vaccination uptake fueled by increased vaccine sensitization and acceptance is essential. High and even vaccine coverage is needed for herd immunity. This is especially important in countries like Brazil, whose government has attracted criticism for handling the pandemic. Increased government support has been associated with the surge in vaccine uptake at the start of 2022. The willingness to vaccinate is not a fixed state and is constantly influenced by information. According to the University of Oxford's *Our World in Data Project*, about 74% of South Americans have been fully vaccinated, which shows good prospects for vaccination [11]. The population must be aware of the inconsistency and risk of protection or immunity imparted by primary infection and the unacceptably high cost of death and morbidity associated with pursuing naturally acquired herd immunity. Continuous reassurance of vaccine safety and effectiveness must be embedded in strategies to boost the uptake of boosters. Even with the uncertainty of herd immunity, the substantial reduction in mortality and morbidity rates will lessen the burden of COVID-19 on the region’s healthcare system.

iii. Vaccine Supply

Increasing vaccine supply improves access, reduces vaccination inequity, especially among the at-risk population, and facilitates vaccine rollout nudges the region towards herd immunity. The deployment of these vaccines necessitates adequate funding for vaccination campaigns and effective data management and prioritizing. Boosting regional capacity to produce vaccines by facilitating technology and knowledge transfer will bridge the inequity gap, expand access, and develop the regions' pharmaceutical manufacturing capacity [29]. This is paramount to promoting health security, nurturing expertise, and ensuring pandemic preparedness [30]. This also contributes to structural transformation and economic growth.

iv. Local vaccine production

The development of policies facilitates the vaccine production journey to support production, the formation of new industrial alliances, and parallel manufacturing by multiple countries and in multiple locations. Global partnerships between governments, domestic producers, international stakeholders and investors, and policies to support supply, all of which are infused with adequate funding and resource utilization, are essential. South America must expand its capacity to develop and produce vaccines. Through collaboration and active support, local production can overcome the hurdles of poor infrastructure, technology inadequacy, and vaccine production skills. Policy framework to strengthen action and development, support equitable vaccine deployment, adequate investment in technology transfer with countries sharing COVID-19-related technologies to enable local production, strategic partnerships, and investments backed by political will. With the success that Cuba has attained with the rollout of Abdala and booster vaccine Soberana Plus, the South American region shows that homegrown vaccines are a critical solution against the pandemic [31]. Taking a cue from the collaborative effort between Argentina and Brazil to produce more doses of the AstraZeneca vaccine, South America’s vaccine pool can grow in efficacy.

e. Population Research

Immunity and the immune responses are complex processes. Several assumptions are made while determining herd immunity and the magnitude of indirect protection provided in a population. The transmission kinetics of the pathogen in the population affected by the epidemic will be a critical consideration. Infectivity is highly reliant on population variables as the accuracy of estimates of the threshold will improve with stable or decreased incidence of the disease [32]. With the evolution in the COVID-19 immunity knowledge, seroprevalence studies on the population are essential to understand how far the region is from the potential for herd immunity. Seroprevalence among South Americans was higher than in Europe, with Peru, Colombia, Argentina, and Brazil recording the highest values [33].

To comprehend the possibility of herd immunity with available vaccines and boosters, as well as to estimate the longevity of SARS-CoV-2 immunity in the wake of recurrent reinfection and the requirements for recurrent vaccinations, critical seroprevalence studies are essential. The continuum of genomic surveillance programs is essential to monitor mutations and the emergence of variants. This population research requires strategic transnational cooperation and knowledge exchange. International partners are integral to building capacity and engagement.

f. Public health confidence

Herd immunity is achievable with the population's steady confidence in the public health system and the vaccination program. It is vital that the cooling of the pandemic and vaccine confidence do not inspire the population to have a relaxed attitude towards the crisis [14]. Even as vaccination has seen increasing population acceptance, developing new variants and waning vaccine immunity should hold the ground for continuous population protection [26]. Herd immunity makes for rare diseases and protects lives and public health measures. While booster dose administration presents a better immune response, it is essential to continue public health measures, especially with actively transmitting infection [34]. Regional and international collaboration for vaccine equity should drive public health interventions for adequate population participation.

g. Vaccination mandates

COVID-19 vaccine mandates across South American countries should be assessed and revised to enable adequate population protection. These provisions and recommendations should immerse population participation to ensure compliance. Vaccination and employment mandates differ across South America, such as in Argentina and Colombia. Employers cannot require employees to be vaccinated, but they can request that they disclose information about their vaccination status voluntarily [35]. Rewards, benefits, and other job incentives can be granted for vaccination. Promoting vaccination should incorporate human rights protection and an equity perspective. Mandates should not violate human rights and should take a much more strategic approach.

## Conclusions

The nuances of the COVID-19 pandemic dictate that achieving herd immunity to COVID-19 requires a changing, adaptive, and flexible perspective with a development stance. Herd immunity is currently not attainable with available vaccines, but hopefully may be in the near future—with more effective vaccines. The waning immunity obtained from available vaccines ensures that present vaccination offers unreliable protection from future waves. This is particularly important with the presence of immune-evasive variants and no ability to predict the properties of future variants. While there is no factual information on the efficacy of future vaccines and the threshold for attaining this crossroad, high and sustained vaccination in the region makes for good coverage. Although herd immunity will have to await for future vaccines, such as the internasal vaccines currently in development, the grounds for population protection covered with available vaccines and public health strategies are commendable. Maintaining a commitment to regional and global solidarity for public security is an essential foundation for vaccine equity. South America’s bid to reach herd immunity should incorporate epidemiological studies, a continuous strong vaccination drive and healthcare provision.

## Data Availability

No database or primary data was used in preparing the manuscript.

## Abbreviations

WHO: World Health Organization
PAHO: Pan American Health Organization
GAVI: Global Alliance for Vaccines and Immunization
